# Ownership and utilisation of long lasting insecticide treated nets following free distribution campaign in South West Nigeria

**DOI:** 10.11604/pamj.2014.17.263.3927

**Published:** 2014-04-11

**Authors:** Sunday Adedeji Aderibigbe, Foluke Adenike Olatona, Oluremi Sogunro, Gafar Alawode, Oluwole Adeyemi Babatunde, Ambrose Itopa Onipe, Oladimeji Akeem Bolarinwa, Hafsat Abolore Ameen, Gordon Kayode Osagbemi, Emmanuel Olatunde Sanya, Adebunmi Oyeladun Olarinoye, Tanimola Makanjuola Akande

**Affiliations:** 1 Department of Epidemiology & Community Health University of Ilorin, Ilorin, Nigeria; 2 Department of Community Health & Primary Care University of Lagos, Lagos State, Nigeria; 3 Yakubu Gowon Centre, Abuja, Nigeria; 4 Department of Community Medicine, Federal Medical Center, Ido, Ekiti State, Nigeria; 5 Department of Medicine University of Ilorin, Ilorin, Nigeria; 6 Department of Obstetrics & Gynaecology University of Ilorin, Ilorin, Nigeria

**Keywords:** Ownership, utilisation, LLIN, South West Nigeria

## Abstract

**Introduction:**

Malaria has proven to be the most horrendous and intractable amongst the health problems confronting countries in the sub-Saharan Africa. This study aims to determine the ownership and utilisation of long lasting insecticide treated nets following free distribution campaign in a state in South West Nigeria.

**Methods:**

Multi-stage sampling technique was used to recruit 2560 households spread across the 16 LGAs of the state. Interviewer administered standardized questionnaire was used for the survey. Data analysis was done using Stata 10 software.

**Results:**

Sixty eight point six percent (68.6%) of the households had at least one under-five child living in the household while 32.6% had at least one pregnant woman living in the household. A total of 2440 (95.3%) households received LLIN during the campaign. Overall, the utilization rate for all respondents was 58.5%. Despite the fact that 2440 households received LLINs during the campaign, only 84.3% of them were seen to have hung theirs during the survey.

**Conclusion:**

Coverage and ownership of LLINs increased significantly following the free distribution campaign. There was a discrepancy between net possession and net use with rate of use lower than possession. Post distribution educational campaign should be incorporated into future distribution campaigns to help increase net utilisation.

## Introduction

Malaria has proven to be the most horrendous and intractable amongst the health problems confronting countries in the sub-Saharan Africa, thereby hampering their development with a high proportion of its wealth being drained by it [1]. There were an estimated 247 million episodes of malaria in 2006, with a wide uncertainty interval (5th - 95th centiles) from 189 million to 327 million cases. Eighty six percent, or 212 million (152-287 million) cases, were in the African Region. Eighty percent of the cases in Africa were in 13 countries, and over half were in Nigeria, Democratic Republic of the Congo, Ethiopia, United Republic of Tanzania and Kenya. There were an estimated 881 000 (610 000-1 212 000) malaria deaths in 2006, of which 91% (801 000, range 52].

The groups most at risk are children under five years of age and pregnant women. Pregnant women are vulnerable because their natural immunity is reduced; thus, they are four times more likely to suffer from complications of malaria than non pregnant women. Malaria is a cause of pregnancy loss, stillbirth, low birth weight, and neonatal mortality. Individuals with sickle cell and other low immune groups are also at higher risk [3].

Malaria negatively impacts the social and economic development of communities in Nigeria. It is responsible for school absenteeism and low productivity at workplaces and on farms. The Federal Government policy on malaria control in Nigeria focuses on the following main interventions: management of cases, prevention of malaria with insecticide-treated nets (ITN), and use of intermittent preventive treatment (IPT) during pregnancy. Presently only 15.5% and 5.4% of rural and urban household in the country has ITN while only 23.2% of under-five children could commence antimalarial drugs immediately after onset of fever [4].

LLIN ownership and use is one of the proven interventions adopted by RBM partners in the country [5]. The target for LLIN coverage and utilisation as contained in the revised NMSP is 100% and 80% respectively i.e. universal coverage. According to the National Implementation Guide for LLIN Distribution, universal coverage translates to ownership of 2 LLINs per household and an assumption of five occupants per household. The use of ITN has been found to reduce clinical malaria by more than 50% and reduces all-cause mortality in children aged 1 - 59 months by 15 - 30% when overall population usage is greater than 70% [5–7]. The objective of this survey was to determine the ownership and utilisation of long lasting insecticide treated nets following a free distribution campaign in a state in South West Nigeria.

## Methods

The study area is one of the thirty-six states in Nigeria. It is a state in southwest Nigeria. The study area has sixteen (16) Local Government Areas. These are Ado-Ekiti, Efon, Ekiti East, Ekiti South-West, Ekiti West, Emure, Ido-Osi, Ijero, Ikere, Ikole, Ilejemeje, Irepodun/Ifelodun, Ise/Orun, Moba, Oye and Gbonyin. The area has a projected population (2009) of 2,612,645 and 522,529 households. It is mainly an upland zone, rising above 250 meters above the sea level. It enjoys tropical climate with two distinct seasons; rainy season (April-October) and the dry season (November-March). Temperature ranges between 21° and 28°C with high humidity. Prior to the LLIN distribution Campaign, the study area had an estimated 6.2% ITN coverage in 2008 based on number of ITNs distributed in the state.

A descriptive cross sectional study design was used in this study. Multi-stage sampling technique was used in view of the large size of the study area. All the sixteen LGAs in the study area were included in the study. The first stage involved a simple random selection of four wards from each of the sixteen LGAs that make up the state. At the ward level, a list of all the settlements that make up each of the selected wards was generated and systematic random sampling was used to select four (4) settlements from each ward. Lastly, a line list of all households in each settlement was also generated and systematic random sampling was used to select ten (10) households from each of the settlements to participate in the survey. The final study population were household heads in 2560 households spread across the 16 LGAs of the state. Interviewer administered standardized questionnaire was used for the survey.

The quantitative data obtained was fed into a computer and analysis was done with STATA version 10. The results were displayed in tables. Cross tabulation of variables were also done. Chi-squared test was used to test for significant associations between variables. A p-value of less than 0.05 was considered as statistically significant.

## Results

Out of the 2560 households surveyed, 68.6% had at least one under-five child living in the household while 32.6% had at least one pregnant woman living in the household. Children under-five years of age and pregnant women accounted for 27.5% and 7.3% of the total population surveyed. Average under-five child per household is 1.4 and average pregnant woman per household is 0.4. Overall, 13,426 persons of all ages were surveyed giving an average household size of 5.2 persons (Table 1).

**Table 1:** Characteristics of the households and respondents surveyed

Variables	Freq (%)
**Households & Respondents Surveyed**	
Households With At Least One Under 5 Child	1757 (68.6)
Households With Two or More Under 5 Children	1064 (41.6)
Households With At Least One Pregnant Woman	835 (32.6)
Households With Two or More Pregnant Women	98 (3.8)
Under 5 Children Surveyed	3688 (27.5)
Pregnant Women Surveyed	978 (7.3)
Others Surveyed	8760 (65.2)
Total Population Surveyed	13426 (100)
Total Households Surveyed	2560 (100)
**Households That Received LLIN**	
Households That Received LLIN (Household Coverage)	2440 (95.3)
Households That Received One LLIN	8 (0.3)
Households That Received Two LLINs	2352 (91.9)
Households That Received More Than Two LLIN	80 (3.1)
Total LLIN Distributed In Surveyed Households	5107 (100)

A total of 2,560 households with 13,426 individuals were surveyed and 5,107 Long Lasting Insecticide Treated Nets were distributed.

A total of 2440 households received LLIN during the campaign out of 2560 households surveyed giving a coverage of 953%. Of the households covered, 0.3% received only one LLIN each, a majority (91.9%) received two LLINs each while 3.1% received more than two LLINs each during the campaign.

More than three quarters of the households (77.1%) did not own a LLIN before the campaign with 3.4% owning two or more LLINs. However, after the campaign, only 4.8% of the households did not have a LLIN with a majority (93.2%) owning two or more LLINs (Table 2). There was a statistically significant difference in the proportion of households with two or more LLIN post campaign (p Usage of LLIN was highest among pregnant women with 88.8% of them sleeping under a LLIN the night preceding the survey (Table 3). Slightly over two thirds (67.5%) of children under-five years of age slept under a LLIN the night preceding the survey while only about half (51.3%) of other members of the household use LLIN. Overall, the utilization rate for all respondents was 58.5%. Despite the fact that 2440 households received LLINs during the campaign, only 84.3% of them were seen to have hung theirs during the survey. There was a statistically significant difference in the proportion of households that have hung their nets when compared to those that have not (Table 4).

**Table 2:** Ownership Of LLIN Among Households After Campaign

Ownership of LLIN	Before Distribution Campaign	After Distribution Campaign	Chi Squared & P value
Households Without LLIN	1973 (77.1)	124 (4.8)	X^2^= 4132.07 p = 0.0000
Households Owning One LLIN	499 (19.5)	50 (2.0)	
Household Owning Two or More LLIN	88 (3.4)	2386 (93.2)	

The distribution campaign involved giving two LLINs to each household that participated in the campaign. Usually all the households in the selected communities are involved

**Table 3:** Utilisation of LLIN by respondents

VARIABLES	FREQ (%)
**Utilisation Among Children < 5. n = 3688**	
Yes	2488 (67.5)
No	1200 (32.5)
**Utilisation Among Pregnant Women. n = 978**	
Yes	868 (88.8)
No	110 (11.2)
**Utilisation Among Others. n = 8760**	
Yes	4497 (51.3)
No	4263 (48.7)
**All Respondents. n = 13426**	
Yes	7856 (58.5)
No	5570 (41.5)

Under Five Children and pregnant women are at greatest risk of developing severe malaria hence utilization is usually measured with these groups in mind.

**Table 4:** Relationship between receiving LLINS and hanging it

Households That Received LLIN	Households Hanging LLIN		Chi Squared & P value
	Yes (%)	No (%)	
Yes	2056 (84.3)	384 (15.7)	X^2^= 276.5 p = 0.0000
No	28 (23.3)	92 (76.7)	

## Discussion

Use of ITN is one of the most cost-effective interventions against malaria [5]. Campaign-like strategies as a means of rapidly increasing LLIN coverage has been embarked on by a number of countries. Since 2002, many countries have begun scaling-up the free or highly subsidized provision of ITNs, including LLINs, and several of them have shown a substantial increase in LLIN coverage as a result [8–12].

In many countries, however, coverage still falls far short of the targets contained in a 2005 World Health Assembly resolution [13], which urged Member States to establish policies and operational plans to ensure that at least 80% of those at risk of, or suffering from malaria should benefit from major preventive and curative interventions by 2010. This survey was carried out in a state in south west Nigeria following one of such free distribution campaigns.

The result showed that prior to the campaign; only 22.9% of the households had treated nets. This corroborates the finding from earlier studies carried out in Nigeria [14] and Uganda [9] in which prior distribution household coverage was 23.9% and 21.9% respectively in some regions of the country. However it is higher than findings from elsewhere in Niger [8] and Ethiopia [15] where prior distribution coverage was 6.3% and 5.3% respectively but lower than findings from another region of Uganda [16] which had 68% prior distribution household coverage.

In all these studies including this present study, the pre distribution coverage rates fell short of the global target of 80% set in a 2005 World Health Assembly resolution [13] and by the Roll Back Malaria partnership [17]. However, this picture of low coverage changed significantly after the campaign in the study area with household LLIN coverage rising to 95.3%. The Niger study [8] also recorded a marked increase from 6.3% to 65.1% coverage rate. It is noteworthy that the high coverage rates recorded were achieved only after free distribution campaigns were initiated in both cases, though the Niger study did not achieve the universal coverage target.

This observation may be a pointer to the fact that for coverage targets to be met at all levels, governments may have to undertake free distribution of nets to households in the community where they reside through campaigns such as was done in this study and not wait for households to take the initiative of purchasing nets on their own. Though it may be argued that for sustainability, government and donor agencies cannot perpetually fund free distribution of nets. However, this assumption is supported by findings from various studies where free distribution has been shown to result in greater ownership, better usage and increased socioeconomic equity in distribution than that achieved by selling LLINS. In Kenya, a comparison of three strategies showed that free mass distribution resulted in a dramatic increase of LLIN ownership and near equality between all socio-economic classes[18]. In Kinshasa, an increase of 54% in LLIN use was seen in women after distribution of free LLINs at the time of delivery [19]. In Tanzania, the largest increases in ownership of LLINs occurred in districts that received free nets during a vaccination campaign [20].

With an average of 5.2 persons per household as recorded in the survey, and the fact that almost all the households received only 2 nets, the issue of universal coverage may yet be a mirage if this trend continues. Sometimes, deciding who sleeps under the net may be a problem that may eventually end up with nobody sleeping under the net [11]. Increasing the number of LLIN given to households to cover all persons living in such households may be the way out but would this be sustainable by government is another issue to contend with.

Ownership of nets does not always translate to usage. This has been demonstrated by authors in previous studies [21–23] where net use does not keep up with possession. The same trend was reported in this survey. Though ownership was as high as 95.3%, only 58.5% of all respondents slept under a LLIN the night preceding the survey. Usage was higher among pregnant women (88.8%) with only 67.5% of children under-five sleeping under a LLIN the night preceding the survey. One reason for this discrepancy as postulated by several authors could be the lack of educational campaigns accompanying LLIN distributions [23–25]. An argument in favour of this position is the increased usage of LLINs recorded in a study in Sierra Leone [12] following a strong educational component of the MSF distribution campaign. Further analysis also revealed that households that received LLIN during the campaign were significantly more likely to retain and hang such nets than those who got their nets from other sources.

Though pregnant women in this study seem to use nets more than other groups and have attained and exceeded the universal coverage target, the goal should still be 100% utilization by all groups. This is because of the degree of morbidity and mortality that can result from malaria infection. Lower LLIN utilization rates when compared with possession may also be a justification for “Hang Up” and “Keep Up” campaigns that have been practiced in other similar surveys [22, 26]. These strategies provide knowledge on the importance of nets and help to demonstrate the proper use of nets immediately following mass distribution. One limitation of this survey is that reasons for not using freely distributed nets were not elicited from the respondents. Otherwise it would have been educative to know why nets are not used despite the fact that they are available. This would have informed interventional strategies to ensure that utilization targets are attained.

## Conclusion

It is concluded that coverage and ownership of LLINs increased significantly following the free distribution campaign surpassing the set universal coverage standard. However, there was a discrepancy between net possession and net use with rate of use lower than possession. Government is encouraged to extend this campaign to other parts of the country in order to achieve the desired universal coverage. Post distribution educational campaign should be incorporated to future distribution campaigns to help increase net utilization.
